# A temporal visualization of chronic obstructive pulmonary disease progression using deep learning and unstructured clinical notes

**DOI:** 10.1186/s12911-019-0984-8

**Published:** 2019-12-17

**Authors:** Chunlei Tang, Joseph M. Plasek, Haohan Zhang, Min-Jeoung Kang, Haokai Sheng, Yun Xiong, David W. Bates, Li Zhou

**Affiliations:** 1 Division of General Internal Medicine and Primary Care, Brigham and Women’s Hospital, Boston, MA USA; 2 Clinical and Quality Analysis, Partners HealthCare System, Boston, MA USA; 3 Shanghai Key Laboratory of Data Science, School of Computer Science, Fudan University, Shanghai, China; 4 School of Computer Science, Carnegie Mellon University, Pittsburgh, PA USA; 5 Loomis Chaffee School, Windsor, CT USA

**Keywords:** “pulmonary disease, chronic obstructive,”, neural networks (computer), Disease progression, Data science

## Abstract

**Background:**

Chronic obstructive pulmonary disease (COPD) is a progressive lung disease that is classified into stages based on disease severity. We aimed to characterize the time to progression prior to death in patients with COPD and to generate a temporal visualization that describes signs and symptoms during different stages of COPD progression.

**Methods:**

We present a two-step approach for visualizing COPD progression at the level of unstructured clinical notes. We included 15,500 COPD patients who both received care within Partners Healthcare’s network and died between 2011 and 2017. We first propose a four-layer deep learning model that utilizes a specially configured recurrent neural network to capture irregular time lapse segments. Using those irregular time lapse segments, we created a temporal visualization (the COPD atlas) to demonstrate COPD progression, which consisted of representative sentences at each time window prior to death based on a fraction of theme words produced by a latent Dirichlet allocation model. We evaluated our approach on an annotated corpus of COPD patients’ unstructured pulmonary, radiology, and cardiology notes.

**Results:**

Experiments compared to the baselines showed that our proposed approach improved interpretability as well as the accuracy of estimating COPD progression.

**Conclusions:**

Our experiments demonstrated that the proposed deep-learning approach to handling temporal variation in COPD progression is feasible and can be used to generate a graphical representation of disease progression using information extracted from clinical notes.

## Introduction

Chronic obstructive pulmonary disease (COPD) is a progressive life threatening lung disease, affecting an estimated 251 million patients globally [1–3]. 5% of all deaths globally are caused by COPD, making it the third leading cause of death [4]. Quality of life deteriorates as COPD progresses from mild symptoms such as breathlessness, chronic cough, and fatigue to serious illness. Death from COPD results most frequently from respiratory failure, heart failure, pulmonary infection, or pulmonary embolism [5]. COPD is not curable [3]. Management of COPD is focused on relieving chronic symptoms, handling exacerbations appropriately, lowering the risk of progression and death, and improving quality of life [3].

The ongoing process of monitoring and assessing a patient’s symptoms and comorbid conditions is essential to effectively managing COPD via appropriate interventions (such as a change in medications). Structured data from clinical research studies is often utilized to study disease progression. For COPD, valuable structured data would include forced expiratory volume in one second (FEV1), forced vital capacity (FVC), the FEV1/FVC ratio, and slow vital capacity (SVC). However, this data may convey an incomplete picture of the patient as these elements may miss critical data stored only in unstructured clinical notes, such as radiology data (e.g., chest X-ray, cardiac radiography) collected for diagnostic and surveillance purposes. Important data for classifying patients to a COPD stage and predicting disease progression may be embedded within these radiology notes and other clinical documents, such as an interpretation of test results and associated clinical findings. Extraction of this knowledge from the electronic health record (EHR) system requires the utilization of data mining and other computational methods [6–8].

There exists a gap in the availability of methods for providing substantial interpretation on the mechanism, progression, and key indicators/measurements for COPD. There are numerous challenges inherent in visualizing COPD progression using large amounts of unstructured clinical documents and classifying these documents into different COPD stages due to:

- *Irregularly sampled temporal data*: Clinical notes are only generated when a patient has a clinical encounter with a clinician at an affiliated medical facility. Thus, the density of relevant clinical documentation in the EHR varies significantly over the span of care for this chronic condition. Although disease progression is a continuous-time process, data for each individual patient is often irregularly sampled due to availability. High density periods may signify the presence of a COPD stage transition as these time periods typically correspond to serious illness. For example, frequent visits or long hospitalizations might indicate a progression whereas less frequent visits may indicate a relatively stable patient state.
- *Individual variability in disease progression*: COPD develops slowly as it often takes ten plus years to evolve from the mild stage to the very severe stage [5]. The rate of disease progression is variable for each individual patient as the primary risk factor is tobacco smoke, thus quitting smoking may delay progression to more severe stages [3]. Conversely, respiratory infections and other exacerbations may move the patient to a more severe stage. Patterns and speed of progression vary across the population.
- *Incompleteness of data*: As COPD is a long term chronic condition, patients may seek COPD care outside of our network.

Modeling a time lapse for each disease stage is the first and foremost step. Utilizing long constant disjoint time windows (e.g., 1 year) may cause issues as that window encompass multiple COPD stages. Short constant disjoint time windows (e.g., 30 days) have been previously utilized by temporal segmentation methods [6] to associate a specific clinical note with its COPD stage. However, constant disjoint time windows cannot adequately represent the dynamics from the temporal autocorrelations that are present.

Capturing the structure of irregular time series data is possible utilizing a recurrent neural network (RNN) [9] or hidden Markov models. RNNs are neural networks with multiple hidden layers where the connections between hidden units form a directed cycle, enabling history to be preserved in internal memory via in these hidden states. RNNs are highly useful in applications where contextual information needs to be stored and updated [10]. Unlike hidden Markov models that are bound by the Markov property where future states depend only upon the present state, not on the sequence of events preceding, RNNs are not bound and can thus keep track of long-distant dependencies. The long-short term memory (LSTM) variant of an RNN is particularly useful as it uses a gated structure to handle long-term event dependencies in order to solve the vanishing and exploding gradient problem. As standard LSTMs cannot handle irregular time intervals [7], prior studies [7, 11] have modified the architecture. Pham et al. [12] solved the irregularly sampled time window issue by setting the forget gate in LSTM to ignore. Similarly, Baytas et al. [7] modified the memory cell of LSTM to account for the elapsed time. The approach of [7, 12] is to adjust the existing data to conform to a regular time interval. Thus, a common limitation of both approaches is that they require that a continuous time hypothesis be formulated [7, 12].

The specific aims of this study were to assess the feasibility (1) in utilizing deep learning to model irregular time segments without the need to formulate a continuous time hypothesis, and (2) of developing a graphical representation (called a COPD atlas) that can visualize and describe COPD conditions during different stages of disease progression in manner interpretable by clinicians and that validly conveys the underlying data.

## Materials and methods

We present a two-step approach for visualizing COPD progression at the level of unstructured clinical notes. First, we developed a four-layer deep learning model extending the LSTM architecture to automatically adjust time interval settings and to represent irregularly sampled time series data. Second, we created a temporal visualization (the COPD atlas) based on those irregular time segments to demonstrate COPD progression. We evaluated the COPD atlas’ performance using human judgement.

### A four-layer model to capture irregular time lapse segments

**Fig 1:**
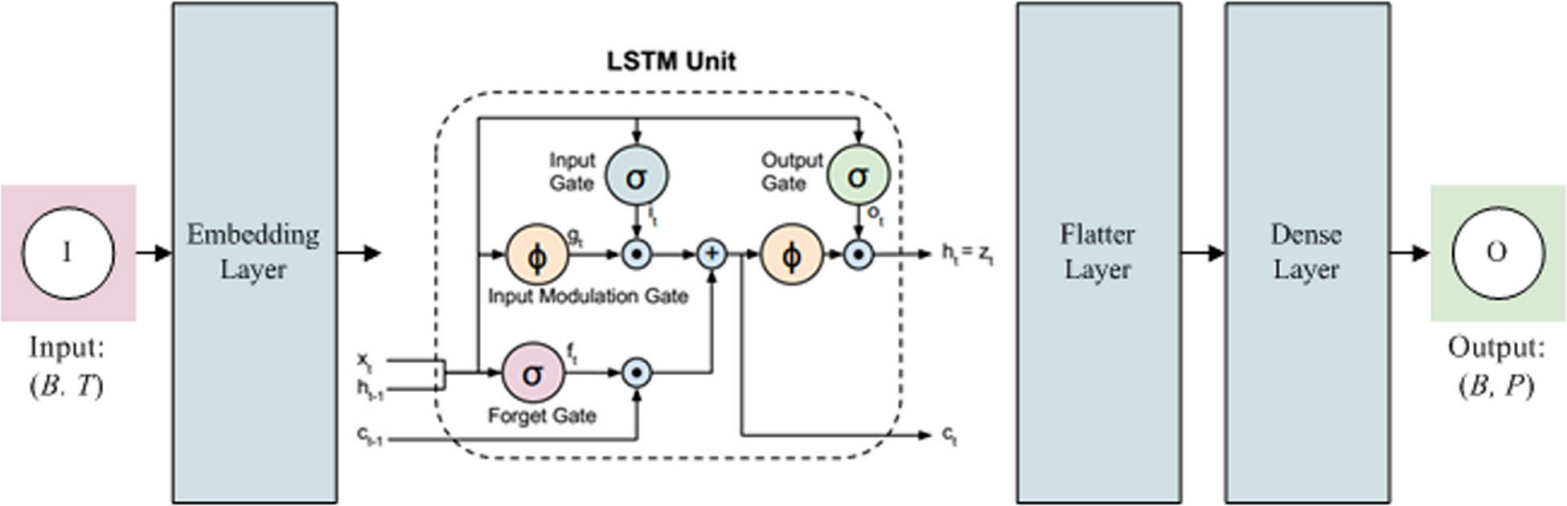
An illustration of the proposed model that includes an embedding layer, long short term memory (LSTM) layer, flatten layer, and dense layer. See Table 1 and Eqs. (1) to (6)

The components of the model include (Fig. 1): 1) a pre-processing and word embedding layer to prepare the data, 2) a LSTM layer to predict death date, and 3) a flatten and dense layer combination to capture the irregular time lapse of segments. An interpretation of notation utilized in this manuscript is available in Table 1. Our model was implemented in Keras (version 2.2.0) on top of Python (version 3.7.0).

**Table 1:** Meaning of notation

Notations	Interpretation
*V*	total number of words in our vocabulary of word embeddings
*v*	dimension of word embeddings in vector space
*T*	maximum words in each input document used for word embeddings
*D*	total number of samples; each sample formed by COPD notes within one day
*B*	Initial quantity of regular time interval used in LSTM
*N = D/B*	total number of batches
*L*	number of hidden units in the LSTM cell
*P*	number of predicted classes

### Pre-processing and word embeddings

A one-hot encoding enables categorical data to have a more expressive representation. We created one-hot encodings of a given regular time interval B for each sample (i.e., input data) to as a pre-processing step. The second step in the pre-processing pipeline utilized Keras padding to ensure that all input samples are the same length and to remove excess data unrelated to COPD. The third step in the pre-processing pipeline utilized an embedding layer in Keras as a hidden layer such that the words extracted from the textual data were represented by dense vectors where a vector represents the projection of the word in continuous vector space. A pre-requisite of this embedding layer is that the input data is integer encoded such that each word is represented by a unique integer. We initialize the embedding layer with random weights. Based on a preliminary analysis of the length and focus of the COPD notes, we defined an embedding layer with a vocabulary V of 10,000, a vector space v of 64 dimensions in which words will be embedded, and input documents T that have 1000 words each. The output of the preprocessing pipeline is an embedding with a dimensionality of (*B*, *T*).

### Long short-term memory unit

LSTMs are well-suited to the task of making predictions given time lags of unknown size and duration between events. The standard LSTM is comprised of input gates, forget gates, output gates, and a memory cell. This standard architecture has the implicit assumption of being uniformly distributed across the elapsed time of a sequence. Detailed mathematical expressions of the LSTM used are given below, in which (1) to (6) are the input gate, forget gate, output gate, input modulation gate, current memory, and current hidden state, respectively (Fig. 1). The output of the LSTM Layers have dimensionality of, (*B*, *T*, *v*), (*B*, *T*, *L*), (*B*, *T × L*), and (*B*, *P*), and are intermediate results from our model. For the dense layer, we can estimate a patient’s mortality if we specify *P* = 1 as the output. Each LSTM matrix is the output from one batch of the period.

1 $$ {i}_t:= \mathrm{sigmoid}\left({W}_{h_i}\times {h}_{t-1}+{W}_{x_i}\times {x}_t+{b}_i\right) $$

2 $$ {f}_t:= \mathrm{sigmoid}\left({W}_{h_f}\times {h}_{t-1}+{W}_{x_f}\times {x}_t+{b}_f\right) $$

3 $$ {o}_t:= \mathrm{sigmoid}\left({W}_{h_o}\times {h}_{t-1}+{W}_{x_o}\times {x}_t+{b}_o\right) $$

4 $$ {g}_t:= \tanh \left({W}_{h_g}\times {h}_{t-1}+{W}_{x_g}\times {x}_t+{b}_g\right) $$

5 $$ {c}_t:= \left({f}_t\cdot {c}_{t-1}\right)+\left({i}_t\cdot {g}_t\right) $$

6 $$ {h}_t:= {o}_t\cdot \tanh {c}_t $$

### Capturing of time lapse segments

To capture irregularly sampled time windows, we used a flatten layer to facilitate the unfolding process followed by a dense layer to combine the time segments into a fully-connected network. We then used a sigmoid activation function for each LSTM matrix to output a sequence (whose dimension is 1) consisting of 0 and 1 as the irregular time lapse segments. Next, iterative learning occurred along the descending direction of gradient descent via the loss function.

Pseudocode is presented below.

### Two baselines for prediction accuracy

We compared performance of the LSTM-based model on the standard metrics against two baseline classifiers: linear regression (LR) and support vector machines (SVMs). Partitioning the time dimension is a linear segmentation problem. We considered different settings for the initial size of the time segments hyperparameter in our proposed model of 30 days, 90 days, and 360 days.

We evaluated our model using a corpus of real-world COPD patient’s clinical notes using 70:30 ratio between the training set and held-out evaluation set. We evaluated our model using standard performance metrics: positive predictive value, and prediction accuracy. We estimate the risk of death in patients using our LSTM-based model on the held-out evaluation dataset using a given clinical note to predict risk of death within a specified period (e.g., 30 days). We calculated positive predictive value of the baselines as the standard for judging whether obtaining irregularly sampled time window from the model is correct or not. Prediction accuracy for the LSTM-based model is calculated as means of comparison between the SoftMax output (which returns a date range corresponding to the predicted patient death date based on one sample) and a patient’s actual death date. Prediction accuracy for LR and SVM was calculated as follows, for each given clinical note: if the absolute difference between the predicted death date from the model and the actual death date is within a given time window set the positive predictive value to 1, else the value is 0.

### Baseline for COPD atlas

Our regional classifier utilizes a spiral timeline to visualize data by presenting topic words identified via latent Dirichlet allocation (LDA) under different themes in a spiral map to show the chronological development of focused themes [13]. To enhance interpretability of our themes, we utilized a representative sentence instead of theme words. More specifically, a representative sentence can be generated by comparing whether the sentence has 3–4 theme words (e.g., 30% of an average sentence length if the entire sentence has 10–14 words) that belong to a specific topic identified by LDA. A spiral timeline is an ideal representation for disease progression as it 1) compactly displays the longest possible length of time in a limited space and 2) avoids having a situation where a correlation between two parallel events is missed if all comparable parameters are similar. Combining timelines with a geographical map enables the depiction of temporal patterns of events with respect to spatial attributes [14]. We utilize the regional classifier as a baseline because it only considers windows of equal-time (e.g., year) rather than irregular time windows, thus enabling us to determine the impact of irregularly sampled time windows for this task. The goal is to compare the top *k* representative sentences captured by the regional classifier to our LSTM-based model to determine this impact on the pulmonary notes’ corpus.

We manually constructed a condensed COPD atlas with the top *k* (=10) representative sentences and invited a panel of subject matter experts consisting of 3 physicians to assist with the evaluation. Our evaluation consisted of two steps: 1) we selected the most recent *n* (=7) enlarged time segments related to the periods prior to death; 2) we generated a list of the top *k* (=10) representative sentences for each time segment.

## Results

### LSTM prediction accuracy at mutiple epochs on merged reports

Our modified LSTM model outperformed the SVM and LR; for example, it achieved a prediction accuracy of 78.85% on our corpus when setting 30 days as the initial size of the temporal segment, compared to the baselines of 8.33 and 0.35% corresponding to SVM and LR, respectively (Table 2).

**Table 2:** LSTM prediction accuracy compared to the baselines

COPD Reports	Initial Size of Time Segment	Prediction Accuracy (%)		
		Our proposed four-layer model	SVM	LR
Merged Reports	30-day	78.85	8.33	0.35
	90-day	80.66	15.38	0.00
	360-day	99.995	33.33	0.00

**Fig 2:**
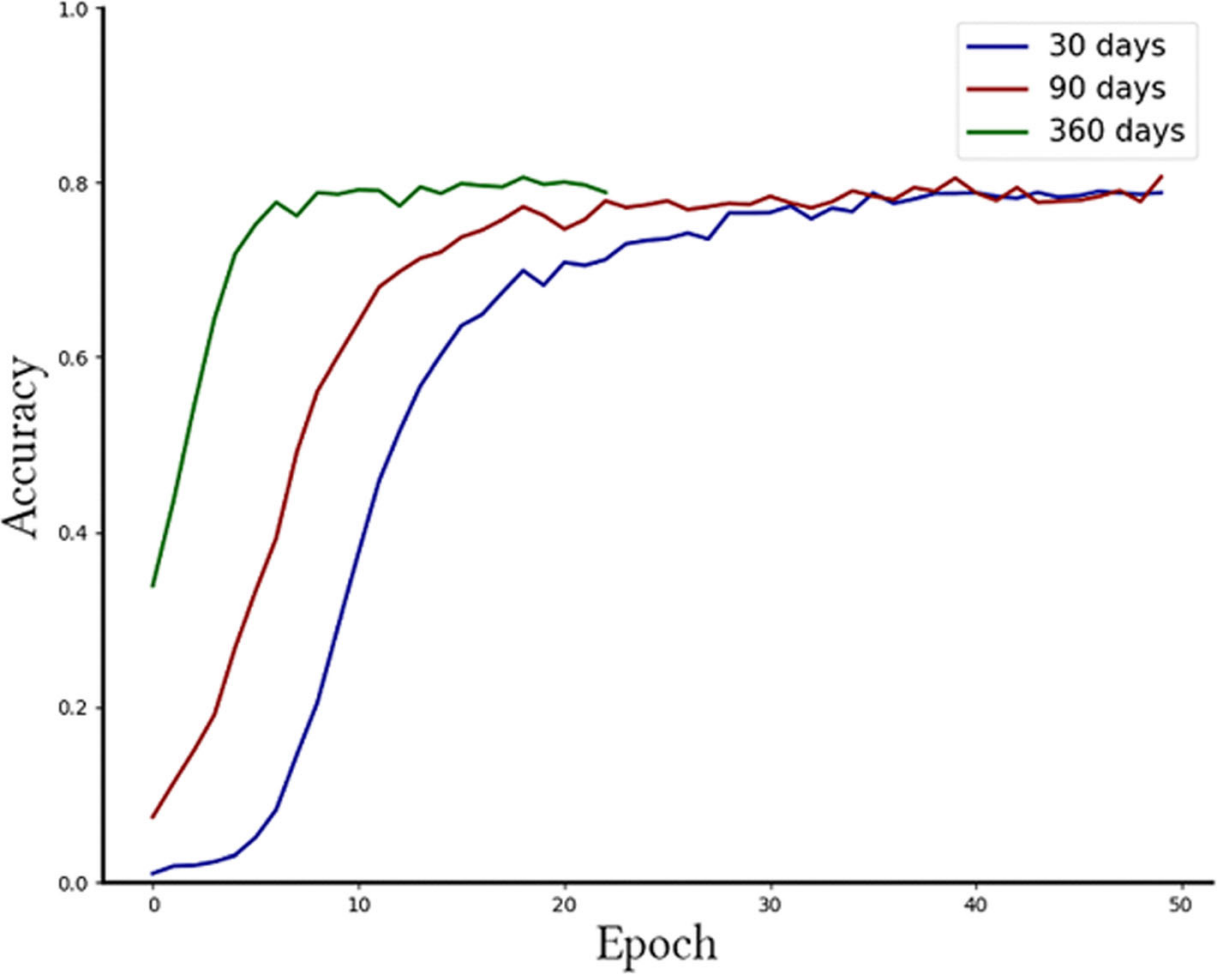
LSTM Prediction accuracy along a sufficient number of epochs

Figure 2 indicates that the initial size of temporal segment is inversely proportional to the number of training epochs. With the window hyperparameter set to 360 days, our model converged in 23 epochs.

### A visualization of the most recent seven time-lapse segments prior to death date on the spiral timeline

**Fig 3:**
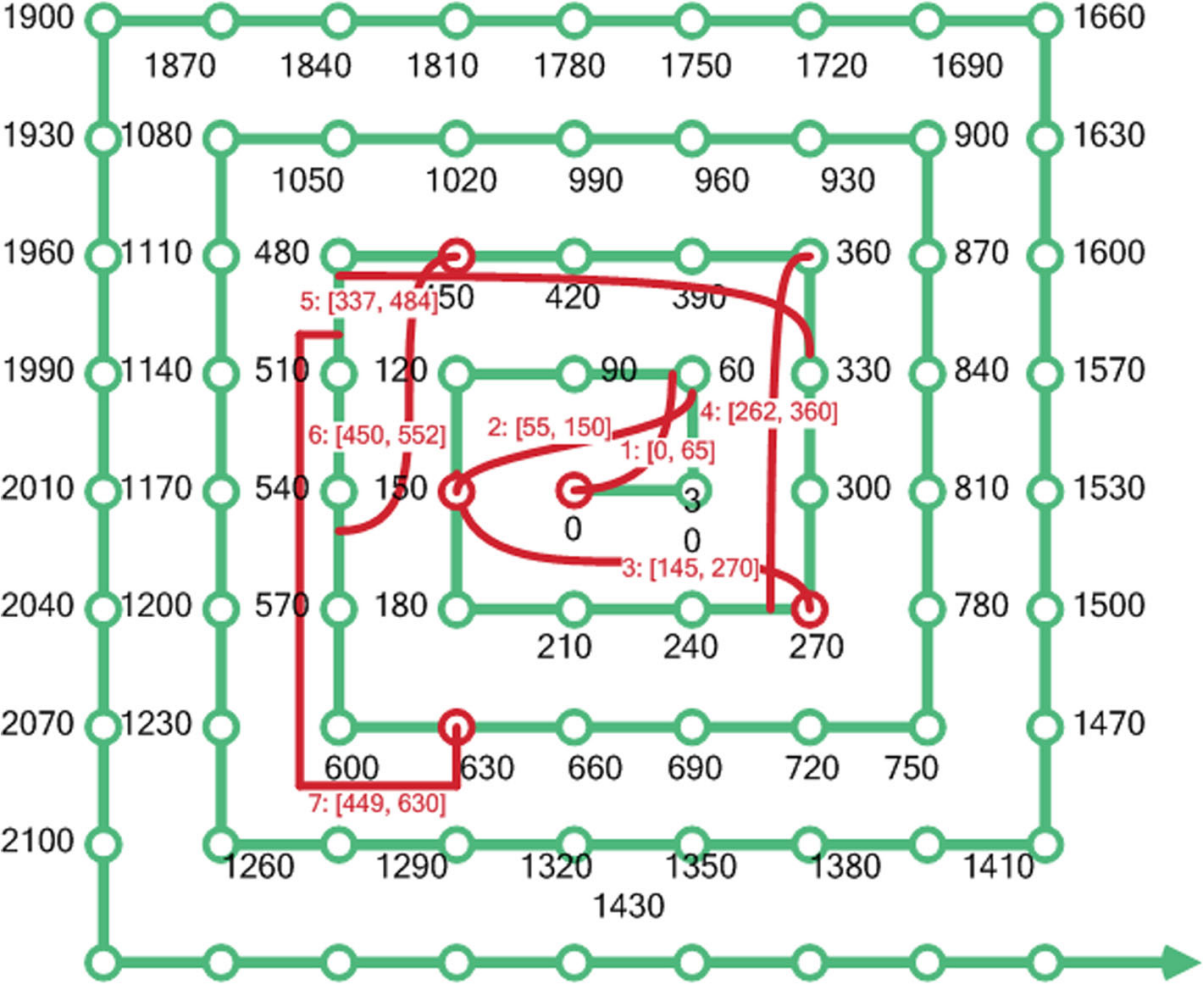
Visualization of the Regional Classifiers standard spiral timeline (i.e., green line with an initial 30-day time window) compared to the first seven irregular time lapse segments (i.e., red line) from our proposed model

Based on the 50 epochs, we obtained a sequence of time lapse segments from the corpus of pulmonary notes using 90 days as the initial size for each time segment. As shown in Fig. 3, we illustrated the most recent seven time-lapse segments prior to death date.

### The COPD atlas generated from pulmonary notes

**Fig 4:**
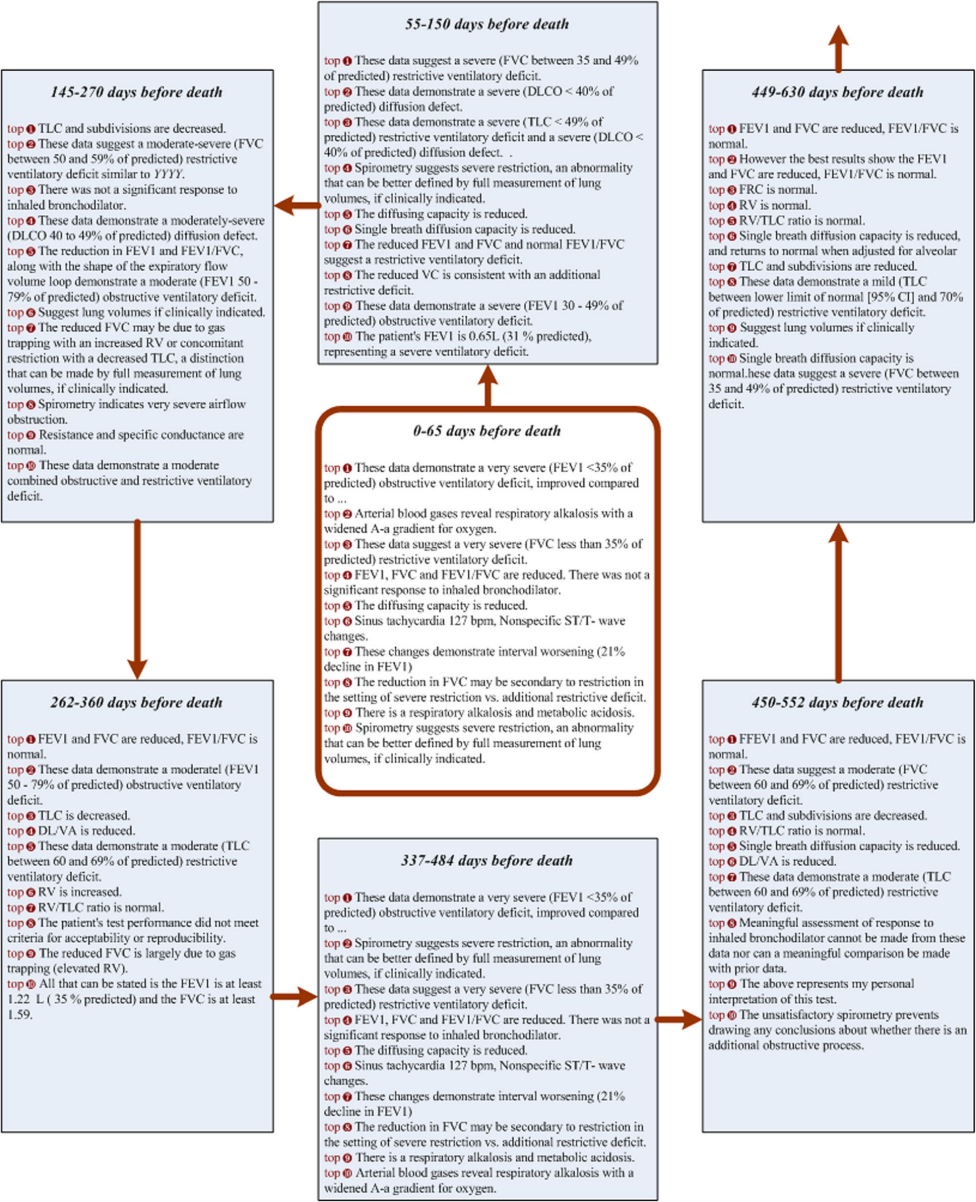
COPD atlas generated from pulmonary notes in the most recent seven time segments prior to death

According to the first seven prior to death captured by our deep learning method, we constructed a condensed COPD atlas using a subset of the identified representative sentences (Fig. 4) Our annotators compared the insights generated from the COPD atlas against the gold version of GOLD criteria, and found that this fluctuating pattern can be utilized by physicians to detect the point at which patients begin to deteriorate and where action may be taken to slow progression. Second, physicians should focus on controlling complications (e.g., heart failure representative sentence #6: “Sinus tachycardia 127 bpm, Nonspecific ST/T- wave changes” was found in the [0–65] day window before death).

## Discussion

The main findings of this study were the establishment of feasibility for our LSTM-based model to predict COPD progression without needing to formulate a continuous time hypothesis, and for generating a COPD atlas. The time windows produced by our LSTM-based model were more interpretable, accurate, and reliable in estimation of COPD mortality compared to baseline methods. Further, our model was found to be robust to the size of the initial time window.

The ability to effectively and efficiently convey detailed information related to disease progression for a particular patient represents an unmet need for chronic diseases (such as COPD, Alzheimer’s, and diabetes) as it could be helpful in informing therapeutic and disease management decisions. This deep learning-based method not only helps us elicit important information regarding progression stage or timing but also is a potentially useful clinical enhancement to generate the COPD atlas. The updated 2018 GOLD guideline uses a combined COPD assessment approach to group patients according to symptoms and their prior history of exacerbations [2]. A COPD atlas enhanced with additional potentially relevant data (such as symptoms, hospitalization history, or additional clinical note types) could then be used for predictive modeling of COPD progression that can then be used to inform COPD guideline modifications. Future telemedicine workflows, patient diaries, and monitoringOther potential clinical applications of the COPD atlas (and potentially a generalized clinical atlas) include: the simultaneous prediction of survival probabilities, signs of developing related diseases, and symptom-associated evolutionary trajectories at different stages of disease progression. The atlas may also address the proxy problem – to predict the probability of death for a given patient within a permissible tolerance range, and to help make recommendations for palliative care referral.

Our approach may be applicable in the palliative and hospice care settings to assist clinician decision making regarding the application of palliative and hospice care to terminal COPD patients. The severe stages of COPD manifest as a lack of physical, social, and emotional functioning, which directly degrade quality of life. In the moderate to severe stages, terminal COPD patients suffer from extreme dyspnea and shortness of breath. 90% of COPD patients suffer from anxiety or depression [14], indicating that COPD patients require emotional support and treatments to relieve the symptoms from COPD related pain. Palliative care and hospice care do improve end-stage patient’s quality of life. However, there often exists a mismatch between patients’ desired and received care at the end of life. In the United States, up to 60% of deaths happen in acute care facilities where patients receive aggressive end of life care due to physicians’ tendencies to over-estimate prognoses and/or their ability to treat the patient [15]. Our research may help reduce physician over-estimates of prognosis and may be instrumental as a decision aid for terminal COPD patients in palliative or hospice care settings.

Our study provides new insights into the visualization of disease progression by investigating methods for general clinical notes corpora instead of the patients who are carefully chosen from clinical trials. This approach makes it much easier to abstract knowledge from clinical practice for use in clinical research. Compared with other studies, our approach combines clinical experience with machine learning. Specifically, selecting the pre-set time windows to partition disease progression comes from physician experience; meanwhile a machine learning approach is utilized to adjust (enlarge) these pre-set time windows by merging clinical notes via the similarity of their content. Considering the frequency of sentence representatives based on the native output of latent Dirichlet allocation (an alternative to embedding or word sense disambiguation techniques) is ingenious but straightforward. Most deep learning embedding approaches require expensive operations (like running a convolutional neural network) to generate (often uninterpretable) representations.

As pulmonary, cardiology, and radiology notes for a patient from the same date may have different correlations to different stages of COPD progression, merging them together using a heuristic merger that does not consider these relationships may not be ideal. This limitation to our study could be mitigated by applying learning methods that compute a score to balance the differences (e.g., priority, dataset size) amongst the three domains. Another limitation is that further research on the COPD atlas is needed to more fully describe each sub-stage clinical characteristics that capture the entire patient experience rather than just what is in the pulmonary notes. For example, although we used clinical reports from multiple domains, we didn’t consider the potentially complex relationships among corpora nor any structured clinical data (e.g., symptoms documented in the problem list of the EHR).

## Conclusions

We developed a novel two-step approach to visualize COPD progression at the level of clinical notes utilizing a four-layer LSTM-based model to capture irregularly sampled time windows. The main findings of this study were the establishment of feasibility for our LSTM-based model to predict COPD progression without needing to formulate a continuous time hypothesis, and for generating a COPD atlas. We addressed a gap in the literature related to the need to formulate a continuous time hypothesis for modeling irregularly sampled time windows. The COPD atlas based on our results produced insightful, interpretable, and reliable results.

## Appendix

The data used in this study is real-word chronic obstructive pulmonary disease corpus and consists of three types of free-text clinical notes (i.e., pulmonarynotes, radiology reports, cardiology reports), which were extracted from the Research Patient Data Registry at Partners Healthcare, an integrated healthcare delivery network located in the greater Boston area of Massachusetts. We retrieved patients’ death dates from Massachusetts Death Certificate files. A cohort of 15,500 COPD patients who both received care at any Partners Healthcare facility and died between 2011 and 2017 was extracted. This study was approved by the Partners Institutional Review Board (IRB).

- *Pulmonary notes*: We extracted physician's interpretation of patients’ lung function from pulmonary notes. Each pulmonary note contains indicators for measuring the air movement in and out of the lungs during respiratory maneuvers (e.g., FVC, FEV1, the FEV1/FVC ratio), as well as a PHYSICIAN INTERPRETATION section. A total of 78,489 pulmonary notes corresponding to 2,431 unique patients were extracted. The average time span of a patient for the pulmonary data source was 724.4 days, with a max span of 3,003 days.
- *Radiology reports*: We extracted chest X-ray radiology reports and focused on two main sections of each report: FINDINGS and IMPRESSION. In our cohort, we had 1,893,498 radiology reports corresponding to 13,414 unique patients. The average time span of a patient using the radiology data source was 843.8 days, with a max span of 2,469 days.
- *Cardiology reports*: We utilized abnormal electrocardiogram reports, and their corresponding patient ID, date of test, and last test date. In our cohort, we had 1,029,363 cardiology reports for 13,918 patients. The average time span of a patient using the cardiology data source was 740.8 days, with a max span of 2,459 days.

## Data Availability

Our research data (i.e., the corpus of clinical notes) is unavailable for access because it is confidential, and it would be cost prohibitive to sufficiently de-identify such a large corpus of clinical documents to remove all patient identifying data according to the HIPAA standard.

## Abbreviations

COPD: Chronic obstructive pulmonary disease
EHR: Electronic health record
FEV1: Forced expiratory volume in one second
FVC: Forced vital capacity
LR: Linear regression
LSTM: Long-short term memory
RNNs: Recurrent neural networks
SVC: Slow vital capacity
SVMs: Support vector machines
